# Effect of the Coronavirus Disease 2019 Pandemic on Retinal Artery Occlusion

**DOI:** 10.1155/joph/5740618

**Published:** 2026-05-05

**Authors:** Tetsuya Muto, Shigeki Machida, Shinichiro Imaizumi, Tetsuju Sekiryu

**Affiliations:** ^1^ Department of Ophthalmology, Imaizumi Eye Hospital, Koriyama, Japan; ^2^ Department of Ophthalmology, Dokkyo Medical University Saitama Medical Center, Koshigaya, Japan, saitama-med.ac.jp; ^3^ Department of Ophthalmology, Fukushima Medical University, Fukushima, Japan, fmu.ac.jp

**Keywords:** best-corrected visual acuity, branch retinal artery occlusion, central retinal artery occlusion, coronavirus disease 2019, prevalence

## Abstract

**Purpose:**

To determine the prevalence and clinical characteristics of retinal artery occlusion (RAO), including branch RAO (BRAO) and central RAO (CRAO), among new patients before and after the declaration of a state of emergency due to coronavirus disease 2019 (April 7, 2020) in Japan.

**Methods:**

New patients and patients with newly diagnosed RAO were categorized into “Before” and “After” groups based on the initial visit. The prevalence, sex ratio, and age of patients newly diagnosed with RAO were compared between the groups. The rates of patients newly diagnosed with rhegmatogenous retinal detachment (RRD) and pseudoexfoliation glaucoma (PEX) were also examined in the Before and After groups.

**Results:**

This study included 53 and 31 patients with BRAO and 55 and 40 patients with CRAO in the Before and After groups, respectively. There were no significant differences in prevalence of RAOs among newly diagnosed patients (BRAO: *p* = 0.449; CRAO: *p* = 0.060), patients with RRD (BRAO: *p* = 0.425; CRAO: *p* = 0.082), or patients with PEX (BRAO: *p* = 0.317; CRAO: *p* = 0.072), as well as in sex ratio and age. Although initial best‐corrected visual acuity (BRAO: *p* = 0.303; CRAO: *p* = 0.291) of RAOs showed no significant differences, the final best‐corrected visual acuity in BRAO significantly worsened in the After group (*p* = 0.002).

**Conclusions:**

Although the coronavirus disease 2019 pandemic did not affect the prevalence of RAOs among new patients compared with that of RRD and PEX, it may have affected its pathophysiology.

## 1. Introduction

The coronavirus disease 2019 (COVID‐19) caused by the severe acute respiratory syndrome coronavirus 2 (SARS‐CoV‐2) is one of the worst pandemics of humankind [1], which resulted in confusion and major changes worldwide. The World Health Organization declared a public health emergency of international concern for COVID‐19 on January 30, 2020, and recommended a policy to prevent the transmission of infection [2].

SARS‐CoV‐2 is an enveloped, plus‐stranded RNA virus with a single‐stranded RNA genome of approximately 30,000 nucleotides [3]. Initially, it was speculated that SARS‐CoV‐2 caused only respiratory functional disorder. However, several reports have confirmed that COVID‐19 caused thrombosis during the course of infection [4–7].

Vaccine development had progressed rapidly to prevent additional COVID‐19 expansion. Between the end of 2020 and 2021, both mRNA vaccines produced by Pfizer/BioNTech and Moderna and adenovirus vector vaccines produced by AstraZeneca and Janssen Pharmaceuticals had been used worldwide; to date, more than 100 vaccines have been developed [8]. In February 2021, mRNA vaccination was initiated in Japan, with cases of COVID‐19 vaccine‐induced thrombosis being reported thereafter [9, 10].

SARS‐CoV‐2 binds to angiotensin‐converting enzyme 2 (ACE2) receptors, which are highly expressed on endothelial cells. In addition, severe COVID‐19 is associated with hyperinflammatory responses. Pathogenesis may lead to an increase in vascular disease clinically [11]. The association of COVID‐19 with vasculitis may cause vascular events in the patients in the ophthalmology clinic.

Both retinal vein occlusion (RVO) and retinal artery occlusion (RAO) are common thrombotic diseases in the ophthalmological setting [12, 13]. The occurrence rate of RVO and RAO might have increased because COVID‐19 itself and its vaccine occasionally lead to the development of thrombosis. Surgery for rhegmatogenous retinal detachment (RRD) and pseudoexfoliation glaucoma (PEX) was selected as a control for comparison. To the best of our knowledge, no reports have indicated that the occurrence rates of surgery for these two diseases were affected by the COVID‐19 pandemic. The objective of this study was to investigate the occurrence rate and pathophysiology of branch RAO (BRAO) and central RAO (CRAO) before and after the declaration of a state of emergency (April 7, 2020) in Japan.

## 2. Materials and Methods

In this study, we retrospectively evaluated 95 patients with naïve CRAO and 84 patients with naïve BRAO diagnosed at Dokkyo Medical University Saitama Medical Center (Koshigaya, Japan) and Fukushima Medical University (Fukushima, Japan) between April 2015 and December 2022. During the same period, 44,967 new patients, 264 with RRD and 116 with PEX, were also evaluated. Patients with symptoms lasting less than 1 month before examination and a history of more than two medical examinations after treatment were included. The CRAO and BRAO diagnosis was based on fundus examination and fluorescein angiographic findings, determined by one of the investigators (Tetsuya Muto). RRD cases were limited to those undergoing scleral buckling surgery as a first‐time procedure. Similarly, PEX cases were limited to those undergoing trabeculectomy or receiving Ex‐Press® (Alcon Laboratories, Fort Worth, TX, USA) implant as a first‐time surgery.

New patients and those with newly diagnosed CRAO, BRAO, RRD, and PEX were categorized into “Before” and “After” groups. CRAO and BRAO were categorized based on the initial diagnosis and the declaration of the state of emergency, while RRD and PEX were categorized based on the initial surgery and the state of emergency. Prevalence rates were compared between the two groups.

We excluded patients with undetermined symptom onset, symptom duration of more than 1 month before examination, senile dementia, complication of RVO, and dense cataracts resulting in poor image quality. Patients with a history of vitrectomy were also excluded. However, patients with pseudophakia who had undergone uncomplicated cataract surgery more than 3 months prior were included.

This study adhered to the tenets of the Declaration of Helsinki. The Institutional Review Board of Dokkyo Medical University Saitama Medical Center and Fukushima Medical University approved the study protocol. Due to the retrospective nature of the study, the need for informed patient consent was waived by the Ethics Committee of both institutions.

At the initial visit, all patients underwent comprehensive ophthalmologic evaluations, including standardized refraction, measurement of best‐corrected visual acuity (BCVA) using a Landolt ring, slit‐lamp biomicroscopy, optical coherence tomography, and color fundus photography. BCVA was expressed as the logarithm of the minimal angle of resolution (logMAR). Patients with BCVA of counting fingers were assigned a logMAR value of 2.6, hand motion value of 2.7, light perception value of 2.8, and no light perception value of 2.9 [14]. Ophthalmologic evaluations, including BCVA, slit‐lamp biomicroscopy, and OCT, were performed, with fluorescein angiography conducted as required. Retinal photocoagulation was performed in cases of vast nonperfusion areas observed on fluorescein angiography.

All data are expressed as the mean ± standard deviation. A stepwise multivariable analysis with a *p* value threshold of 0.2 was used to identify the factors associated with the final BCVA. Factors included sex, age, laterality, initial BCVA, duration from onset to initial visit, ocular massage, oral administration of kallidinogenase, eye solution for glaucoma, combination of neovascular glaucoma (NVG), intravenous drip of vasodilator or thrombolytic agent, and oral administration of anticoagulant. Statistical software included StatMate® version V for Macintosh (ATMS, Tokyo, Japan) for unpaired *t* tests and chi‐squared tests and JMP 16 Pro (SAS Institute, Inc., Cary, NC, USA) for multivariable analysis. The chi‐squared test was used to determine the statistical significance of the prevalence rates, sex ratio, laterality ratio, NVG, blindness rates, and better visual prognosis rates. An unpaired *t*‐test was used to compare the groups before and after the state of emergency. Differences with a *p* value < 0.05 were considered statistically significant.

## 3. Results

Table 1 shows patient demographics of BRAO and CRAO in the Before and After groups. No significant differences were found in prevalence rates, sex ratio and age between the Before and After groups of RAOs among newly diagnosed patients, patients with RRD, or those with PEX.

**TABLE 1:** Patient demographics of BRAO and CRAO in the Before and After groups.

	Before group	After group	*p* value
Patients newly diagnosed with BRAO/new patients	53/30122	31/14845	0.449
Patients newly diagnosed with CRAO/new patients	55/30122	40/14845	0.060
Patients newly diagnosed with BRAO/patients newly diagnosed with RRD	53/179	31/85	0.425
Patients newly diagnosed with CRAO/patients newly diagnosed with RRD	55/179	40/85	0.082
Patients newly diagnosed with BRAO/patients newly diagnosed with PEX	53/81	31/35	0.317
Patients newly diagnosed with CRAO/patients newly diagnosed with PEX	55/81	40/35	0.072
Sex of patients with BRAO (male/female)	33/20	17/14	0.503
Sex of patients with CRAO (male/female)	30/25	29/11	0.075
Age of patients with BRAO (years)	69.45 ± 13.51	67.87 ± 19.16	0.687
Age of patients with CRAO (years)	69.73 ± 14.86	69.53 ± 14.65	0.948

Abbreviations: BRAO, branch retinal artery occlusion; CRAO, central retinal artery occlusion; PEX, pseudoexfoliation glaucoma; RRD, rhegmatogenous retinal detachment.

Table 2 shows the characteristics of 84 eyes of BRAO and 95 eyes of CRAO in the Before and After groups. No significant differences were observed in all factors except the time between the initial visit and symptom onset (*p* = 0.033) and final BCVA (*p* = 0.002) in BRAO. Time was shorter and the final BCVA was poorer in the After group compared with the Before group.

**TABLE 2:** Characteristics of BRAO (84 eyes) and CRAO (95 eyes) in the Before and After groups.

	Before group	After group	*p* value
Time between initial visit and symptom onset in BRAO (days)	4.91 ± 6.45	2.68 ± 2.90	0.033
Time between initial visit and symptom onset in CRAO (days)	3.07 ± 5.85	3.10 ± 6.50	0.983
Initial BCVA in BRAO	0.49 ± 0.94	0.85 ± 0.33	0.303
Initial BCVA in CRAO	1.80 ± 1.03	1.99 ± 0.89	0.291
Laterality in BRAO (right/left)	34/19	15/16	0.157
Laterality in CRAO (right/left)	24/31	19/21	0.709
Outbreak of NVG in BRAO (yes/no)	1/52	0/31	0.442
Outbreak of NVG in CRAO (yes/no)	2/53	2/38	0.744
NLP/more than LP after treatment in BRAO	4/49	0/31	0.117
NLP/more than LP after treatment in CRAO	10/45	3/37	0.233
Final BCVA in BRAO	0.68 ± 1.11	0.80 ± 0.39	0.002
Final BCVA in CRAO	1.54 ± 1.05	1.65 ± 1.02	0.647
Final BCVA less than zero in BRAO (yes/no)	29/24	19/12	0.557
Final BCVA less than zero in CRAO (yes/no)	7/48	3/37	0.630

Abbreviations: BCVA, best‐corrected visual acuity; BRAO, branch retinal artery occlusion; CRAO, central retinal artery occlusion; LP, light perception; NLP, nonlight perception; NVG, neovascular glaucoma.

Table 3 shows the factors associated with final BCVA using multivariable analysis of 53 eyes in the Before group and 31 eyes in the After group of BRAO. Initial BCVA (*p* < 0.001) and combination of NVG (*p* = 0.011) were associated with final BCVA in the Before group.

**TABLE 3:** Associated factors with final BCVA of BRAO in the Before and After groups using the multiple logistic regression model with a backward stepwise selection method.

Factors	95% CI	*p* value
*Before group (n = 53)*		
Sex	−0.377 to 0.321	0.871
Age	−0.011 to 0.016	0.731
Laterality	−0.330 to 0.397	0.852
Initial BCVA	0.593 to 0.976	< 0.001
Time between initial visit and symptom onset (days)	−0.032 to 0.022	0.717
Ocular massage	−0.028 to 0.842	0.066
Oral administration of kallidinogenase	−0.335 to 0.510	0.678
Eye solution for glaucoma	−0.243 to 0.689	0.340
Combination of NVG	−2.771 to −0.382	0.011
Intravenous drip of vasodilator or thrombolytic agent	−0.510 to 0.441	0.884
Oral administration of anticoagulant	−0.152 to 0.632	0.223

*After group (n = 31)*		
Sex	−0.092 to 0.472	0.175
Age	0.000 to 0.015	0.041
Laterality	−0.088 to 0.620	0.133
Initial BCVA	0.078 to 0.579	0.013
Time between initial visit and symptom onset (days)	−0.054 to 0.055	0.987
Ocular massage	−0.804 to 0.398	0.489
Oral administration of kallidinogenase	−0.372 to 0.277	0.763
Eye solution for glaucoma	−0.281 to 0.582	0.476
Combination of NVG	0.018 to 1.266	0.044
Intravenous drip of vasodilator or thrombolytic agent	n/a	n/a
Oral administration of anticoagulant	−0.521 to 0.118	0.203

Abbreviations: BCVA, best‐corrected visual acuity; BRAO, branch retinal artery occlusion; CI, confidence interval; n/a, not available; NVG, neovascular glaucoma.

However, age (*p* = 0.041), initial BCVA (*p* = 0.013), and intravenous drip of the vasodilator or thrombolytic agent (*p* = 0.044) was significantly associated in the After group.

Table 4 shows the factors associated with final BCVA using multivariable analysis of 54 eyes in the Before group and 40 eyes in the After group of CRAO. Initial BCVA (*p* < 0.001) was significantly associated in both groups.

**TABLE 4:** Associated factors with final BCVA of CRAO in the Before and After groups using the multiple logistic regression model with a backward stepwise selection method.

Factors	95% CI	*p* value
*Before group (n = 54)*		
Sex	−0.692 to 0.557	0.828
Age	−0.015 to 0.028	0.520
Laterality	−0.801 to 0.543	0.700
Time between initial visit and symptom onset (days)	−0.088 to 0.056	0.654
Ocular massage	−0.722 to 0.991	0.753
Oral administration of kallidinogenase	−0.037 to 1.779	0.060
Eye solution for glaucoma	−0.893 to 1.145	0.804
Combination of NVG	−3.385 to 1.861	0.561
Oral administration of anticoagulant	−0.382 to 1.181	0.308

*After group (n = 40)*		
Sex	−0.631 to 1.127	0.569
Age	−0.025 to 0.024	0.994
Laterality	−0.800 to 0.663	0.849
Time between initial visit and symptom onset (days)	−0.022 to 0.101	0.198
Ocular massage	−1.816 to 0.266	0.010
Oral administration of kallidinogenase	−0.918 to 0.864	0.951
Eye solution for glaucoma	−1.295 to 0.306	0.217
Combination of NVG	−2.607 to 1.150	0.435
Oral administration of anticoagulant	−1.400 to 0.216	0.145

Abbreviations: BCVA, best‐corrected visual acuity; CI, confidence interval; CRAO, central retinal artery occlusion; NVG, neovascular glaucoma.

## 4. Discussion

According to our results, the occurrence rate of RAOs did not change between the two groups.

Table 5 presents a summary of the findings from large‐scale data analyses and systematic reviews on this topic [15–24]. Al‐Moujahed et al. reported an initial increase in CRAO cases during the early months of the pandemic [15]. However, RAO diagnostic rates as a proportion of all new retinal clinic diagnoses remained stable throughout most of the pandemic [15]. Cho et al. reported that the overall incidence of RAO after the COVID‐19 pandemic did not increase; in fact, it exhibited a slight decrease compared with that before the outbreak [16]. Modjtahedi et al. observed no significant change in the RAO incidence related to COVID‐19 [17]. Park et al. found no significant relationship between COVID‐19 infection [18, 19] or vaccination [18] and the incidence of RAO or RVO. Au et al. concluded COVID‐19‐related CRAO cases were just coincidental [20]. Both Yeung et al. and Singh et al. did not mention the correlation between RAO and COVID‐19 vaccination in their reviews [21, 22]. In contrast, Li et al. and Feltgen et al. reported a significant increase in RAO and RVO after COVID‐19 vaccination [23, 24].

**TABLE 5:** Association between RAOs and COVID‐19 pandemic from large‐scale data analyses and systematic reviews.

No.	Authors	Number of patients	Conclusions
1	Al‐Moujahed et al. [15]	442,186 patients	The number of patients with RAO increased during the first few months of the COVID‐19 pandemic.
2	Cho et al. [16]	32,028 patients diagnosed with RAO	The incidence of RAO did not increase during the COVID‐19 outbreak.
3	Modjtahedi et al. [17]	432,515 patients diagnosed with COVID‐19 infection	There was a smaller increase in the incidence of RAOs after COVID‐19 diagnosis
4	Park et al. [18]	8,418,590 patients	No increase in the hazard ratio of RAO relative to COVID‐19 or COVID‐19 vaccination, except for a possible increase in the hazard ratio of RAO among women vaccinated with mRNA‐1273.
5	Park et al. [19]	21,696 patients with RAO	SARS‐CoV‐2 infection did not significantly increase the incidence of RAO.
6	Au and Ko [20]	15 patients with CRAO	COVID‐19 related CRAO cases were just coincident.
7	Yeung et al. [21]	15 patients with RAO	Correlation between RAO and COVID‐19 vaccination was not mentioned.
8	Singh et al. [22]	433 patients with RAO	Correlation between RAO and COVID‐19 vaccination was not mentioned.
9	Li et al. [23]	1,478,132 patients	The risk of RAO and retinal vein occlusion significantly increased during the first 2 weeks after vaccination.
10	Feltgen et al. [24]	421 patients with RAO and retinal vein occlusion	No evidence of any association was found between SARS‐CoV‐2 vaccination and a higher risk for RAO and retinal vein occlusion.

Abbreviations: COVID‐19, coronavirus disease 2019; CRAO, central retinal artery occlusion; RAO, retinal artery occlusion; RVO, retinal vein occlusion; SARS‐CoV‐2, severe acute respiratory syndrome coronavirus 2.

Although the occurrence rate and male rate of CRAO tended to increase in the After group, the differences were barely not significant. The incidence of several diseases, such as acute primary angle closure [25], Vogt–Koyanagi–Harada disease [26], optic neuritis [27], dry eye [28], and infectious endophthalmitis after vitrectomy [29], increased after the COVID‐19 pandemic.

Rubino et al. reported that the prevalence of thromboembolic events was significantly higher in children with multisystem inflammatory syndrome after the COVID‐19 pandemic [30].

Theoretically, it is no wonder that RAOs increase like the above diseases [25–30].

Time between initial visit and symptom onset in BRAO was shorter in the After group compared with the Before group, and we speculated that remote work had enabled workers to visit the hospital. Because workers were easy to use time freely in such situations. However, it had not a significant difference in CRAO, and the reason for this observation was undetermined. Although the time between initial visit and symptom onset was shorter in the After group compared with the Before group, the final BCVA of BRAO was poorer in the former group. Shah et al. reported that a shorter symptom duration before treatment did not influence final visual acuity outcomes in CRAO [31]; this result might be proper.

Initial BCVA and NVG of BRAO were associated with final BCVA in the Before group, while age, initial BCVA, and intravenous drip of vasodilator or thrombolytic agent were associated in the After group. There were no NVG cases observed in the After group; thus, it was not possible to perform statistical analysis. In general, NVG rarely develops in patients with BRAO [32, 33]. However, the development of NVG is associated with poor visual prognosis [34]. Mason et al. reported that visual prognosis after BRAO seems to be correlated to presenting visual acuity [35]. Yuzurihara and Iijima reported that the visual acuity of patients with CRAO is poor at presentation, and the prognosis is generally poor, with a few exceptions [36]. In contrast, the visual acuity of patients with BRAO is markedly better both at presentation and the final visit [36]. In general, arteriosclerosis of young age is milder than in old age, and reopening of the artery occlusion tends to occur. Although we could not find reports regarding the use of an intravenous drip of a vasodilator or thrombolytic agent in BRAO, several studies reported the use of such therapy in CRAO [37–39]. Schrag et al. showed that systemic fibrinolysis is significantly effective only within the first 4.5 h of symptom onset [37]. Janská et al. stated that intravenous thrombolytic therapy in CRAO appears to be effective and safe [38]. Suzuki et al. showed that visual acuity and retinal vein constriction improved after liposomal prostaglandin E1 therapy [39]. Although Yasuda et al. reported that female sex (odds ratio = 15.63, *p* = 0.032) was independently associated with worse visual acuity in BRAO [40], our results did not reveal associations with sex. Analysis of additional data are required to confirm these findings.

In this study, we selected RRD and PEX as control diseases. The prevalence rates of several ocular diseases were affected by COVID‐19 [25–30]. Nevertheless, there were no reports found concerning the change in the prevalence rates of RRD and PEX according to the results of a search in the PubMed database (keywords: “COVID‐19, RRD” or “COVID‐19, buckling surgery” or “COVID‐19, pseudoexfoliation syndrome” or “COVID‐19, glaucoma surgery”; search years: 2000–2024). Particularly, PEX was considered the best control disease because the age of onset of RAOs and the age of PEX that required trabeculectomy or use of an Ex‐Press® implant as a first‐time surgery were almost the same.

This study had several limitations. First, the tendency for an increase in the occurrence and male rates of CRAO in the After group was barely not statistically significant. Analysis of a larger sample could have yielded different results. Second, we only investigated ocular massage in cases with a description on the medical record. It is possible that ocular massage was performed without description in the record. Third, the number of individuals who had COVID‐19 or received vaccination was unclear due to the retrospective nature of the study. Fourth, the detailed mechanism by which these factors might influence the clinical features of RAOs remains unclear.

## 5. Conclusions

Our results show that the prevalence of RAOs did not increase during the COVID‐19 pandemic. However, the clinical features of RAOs may have been influenced mildly by the pandemic.

## Funding

The authors declare that they have received no funding.

## Conflicts of Interest

The authors declare no conflicts of interest.

## Data Availability

The datasets used and/or analyzed during this study are available from the corresponding author upon reasonable request.
